# Prevalence of depression among HIV-positive pregnant women and its association with adherence to antiretroviral therapy in Addis Ababa, Ethiopia

**DOI:** 10.1371/journal.pone.0262638

**Published:** 2022-01-20

**Authors:** Workeabeba Abebe, Mahlet Gebremariam, Mitike Molla, Solomon Teferra, Larry Wissow, Andrea Ruff

**Affiliations:** 1 Department of Pediatrics and Child Health, School of Medicine, College of Health Sciences, Addis Ababa University, Addis Ababa, Ethiopia; 2 Department of Obstetrics and Gynecology, School of Medicine, College of Health Sciences, Addis Ababa University, Addis Ababa, Ethiopia; 3 Department of Preventive Medicine, School of Public Health, College of Health Sciences, Addis Ababa University, Addis Ababa, Ethiopia; 4 Department of Psychiatry, School of Medicine, College of Health Sciences, Addis Ababa University, Addis Ababa, Ethiopia; 5 Department of Psychiatry and Behavioral Sciences, University of Washington, Seattle, Washington, United States of America; 6 Department of International Health, Johns Hopkins University Bloomberg School of Public Health, Baltimore, MD, United States of America; National University of Singapore, SINGAPORE

## Abstract

**Background:**

Vertical transmission of HIV remains one of the most common transmission modes. Antiretroviral therapy (ART) decreases the risk of transmission to less than 2%, but maintaining adherence to treatment remains a challenge. Some of the commonly reported barriers to adherence to ART include stress (physical and emotional), depression, and alcohol and drug abuse. Integrating screening and treatment for psychological problem such as depression was reported to improve adherence. In this study, we sought to determine the prevalence of depression and its association with adherence to ART among HIV-positive pregnant women attending antenatal care (ANC) clinics in Addis Ababa, Ethiopia.

**Methods:**

We conducted a cross-sectional survey from March through November 2018. Participants were conveniently sampled from 12 health institutions offering ANC services. We used the Patient Health Questionnaire-9 (PHQ-9) to screen for depression and the Center for Adherence Support Evaluation (CASE) Adherence index to evaluate adherence to ART. Descriptive statistics was used to estimate the prevalence of depression during third-trimester pregnancy and nonadherence to ART. A bivariate logistic regression analysis was used to get significant predictors for each of the two outcome measures. The final multivariable logistic regression analysis included variables with a *P*<0.25 in the bivariate logistic regression model; statistical significance was evaluated at *P*<0.05.

**Results:**

We approached 397 eligible individuals, of whom 368 (92.7%) participated and were included in the analysis. Of the total participants, 175(47.6%) had depression. The participants’ overall level of adherence to ART was 82%. Pregnant women with low income were twice more likely to have depression (AOR = 2.10, 95%CI = 1.31–3.36). Women with WHO clinical Stage 1 disease were less likely to have depression than women with more advanced disease (AOR = 0.16, 95%CI = 0.05–0.48). There was a statistically significant association between depression and nonadherence to ART (P = 0.020); nonadherence was nearly two times higher among participants with depression (AOR = 1.88, 95%CI = 1.08–3.27).

**Conclusion:**

We found a high prevalence of depression among HIV-positive pregnant women in the selected health facilities in Addis Ababa, and what was more concerning was its association with higher rates of nonadherence to ART adversely affecting the outcome of their HIV care. We recommend integrating screening for depression in routine ANC services.

## Introduction

The human immunodeficiency virus (HIV) remains a significant public health problem in low and middle-income countries (LMICs), especially in sub-Saharan African countries, including Ethiopia. In 2020, 37.7 million people worldwide, including 1.7 million children, were infected with the virus [1]. Ethiopia has achieved a 90% decline in new HIV infection rates in recent years; nevertheless, a 0.12 of new infections per 1000 population of all ages still occur, significant given the large population[2]. According to 2020 estimate, 620 000 individuals live with HIV in Ethiopia, with a national prevalence rate of 1% [2].

Although perinatal transmission continues to result in a high number of HIV-positive children around the world, the use of ART among HIV-infected pregnant women can effectively lower transmission rates to below 2% [3]. In Ethiopia, free ART service was first launched in public hospitals in 2005 and then scaled up to primary health care facilities starting in 2006. In 2013, the Ethiopian government made a commitment to eliminate mother-to-child transmission (MTCT) of HIV. To achieve this, all pregnant women had to be screened for HIV, and those found to be HIV-positive were provided ART (Option B+). Option B+ provides the single-pill triple anti-retroviral therapy (ARV) drugs to all HIV-positive pregnant women, beginning in the ANC setting and continuing this therapy for life without the need for an initial CD4 test. The HIV program provides a complete blood count, CD4, and viral load testing for free for all HIV-positive pregnant women at the first trimester or any time they first present to health institutions [4,5].

For the prevention of mother-to-child transmission (PMTCT) to be successful, a pregnant woman has to take ART consistently until the child is born and while breast-feeding. However, maintaining adherence remains a challenge. A comprehensive review of ART adherence studies involving more than 20,000 women during and after pregnancy in LMICs, including countries from sub-Saharan Africa, found a 73.5% optimal adherence to ART, as defined by greater than 80% adherence. Commonly reported barriers to adherence included were stress (physical or emotional), depression, alcohol, and drug abuse [6]. More recent reviews of patient-reported barriers to adherence also identified depression as a significant factor [7,8], and a meta-analysis of studies from sub-Saharan Africa found a 55% lower rate of adherence to ART among individuals with depression compared to patients without depression [9]. It is important to note that not all studies have found an association between depression and adherence; for instance, a study in Uganda among individuals purchasing ART did not find depression predicted lack of adherence [10]. Similarly, a study from the Democratic Republic of Congo that identified 11.8% of pregnant HIV-positive women in their cohort as depressed did not find an association between depression and loss to follow-up [11].

A systematic review of literature through 2008 evaluating pre-and postnatal maternal mental status among African women, depression found to be the most commonly reported condition. Weighted prevalence rates were 11.3% and 18.3% in the pre-and postpartum populations, respectively [12]. Another systematic review evaluating data from LMICs reported weighted mean of pre-and postpartum depression prevalence rates at 15.6% and 19.8%, respectively [13].

In Ethiopia, the prevalence of common mental disorders among pregnant mothers in a long-standing rural cohort, including depression, was reported to be 5% [14,15], lower than the average for LMICs [13,16]. Sociocultural practices were reported to be protective of these mothers from developing depression [15]. A study conducted in Ethiopia’s Amhara region using Self-reporting Questionnaire (SRQ-20) developed by WHO on 1,319 women who had given birth within the last 24 months reported that 32.9% had probable common mental disorder (CMD), including depression [17]. Two studies in urban settings, one using the Patient Health Questionnaire—9 (PHQ-9) with a cutoff point of 4 or more among 187 pregnant women (10.7% HIV-positive), and the other using Edinburgh Postnatal Depression Scale (EPDS) among 393 women, reported the prevalence of depression 29.9% and 24.94% respectively [18,19].

As stated above, depression has been identified as a barrier to optimal adherence to HIV treatment. HIV infection itself has been associated with an increased prevalence of depression [20]. Although HIV care and ART have been associated with improvements in mental health [21,22], depression remains prevalent. A recent study in Kenya reported that 48% of 123 HIV-positive women eight weeks postpartum had elevated depression scores [23].

Treating depression potentially improves several HIV outcomes, including adherence to ART [24]. To our knowledge, there was no study from Ethiopia that has reported the prevalence of depression among HIV-positive pregnant women and its association with adherence to ART. Thus, studying the prevalence of depression and its association with adherence to ART in Ethiopia was expected to generate evidence to improve the care for people living with HIV and prevent new infections.

## Methods

### Study design and setting

We conducted a cross-sectional study in 12 public health institutions in Addis Ababa (six hospitals and six health centers) selected based on the number of HIV-positive women attending the respective institutions [25]. Addis Ababa has 11 public hospitals and 56 public health centers provide maternity care, including ANC, delivery, and postnatal care. Prevention of mother-to-child transmission (PMTCT) of HIV using Option B+ has been institutionalized in all facilities providing maternal and child health services in Addis Ababa since 2013. Antenatal care and PMTCT services are provided fully subsidized in all public health institutions of Addis Ababa. As part of Option B+, ANC services are provided to all HIV-positive pregnant women monthly till delivery [4].

### Study participants

All HIV-positive pregnant women in their third trimester of pregnancy with a follow-up at the ANC clinic of the selected health institutions were eligible for inclusion in the study. Those who were less than 18 years old, and who lacked documentation of HIV serostatus, were not willing to participate, were not able to provide informed consent, or were acutely ill on the day of the interview (acutely ill, meaning the woman has new clinical symptoms) were excluded from the study.

### Sample size

The sample size of 397 was calculated based on the formula for a single population proportion by assuming a prevalence of depression of 39% [26], 95%confidence level, a margin of error (D) = 5%, Design effect *(Deff)* of 1, and 10% non-response rate.

Our second objective was to assess the association of depression with nonadherence to ART among HIV-positive pregnant women. We assumed that 80%of recruited HIV-infected pregnant women would have good adherence to their ART [6] and that the rate of nonadherence in women with depression would be lower, estimated at 55%, based on a meta-analysis of studies from sub-Saharan Africa [9].

We calculated the total number of subjects needed to demonstrate a difference in proportions of nonadherence among HIV-positive pregnant women with depression compared to HIV-positive pregnant women who do not have depression. The sample size calculated based on a two-proportion formula and anticipating a maximum dropout of 10% was 299 HIV-positive pregnant. This sample size was smaller than that required for the first objective; therefore, the sample size of 397 participants was used. All eligible and consenting pregnant women in their third trimester presenting to the selected health facility during the study period were included until the required sample size was achieved.

### Measurements

#### Data collection tools

A research team developed a structured questionnaire to collect general socio-demographic indicators, clinical information, and substance use. Data on CD4, viral load, and nutritional assessment were extracted from medical records. The questionnaire was prepared in English and then translated into Amharic, the national language of Ethiopia. Independent personnel again back-translated the tool into English to ensure consistency, and the tool was pretested among ten pregnant women attending ANC clinic at a hospital.

#### Screening for depression

The Patient Health Questionnaire -9 (PHQ-9), a brief screening instrument for depression, was used to collect data on depression [27]. In Ethiopian adults, the PHQ-9 was found to be a reliable and valid instrument. The r score’s reliability was Cronbachs α  =  .85 and test re-test reliability (intraclass correlation coefficient = 0.92). A factor analysis confirmed a 1-factor structure. Receiver Operating Characteristics (ROC) analysis showed that a PHQ-9 threshold score of 10 offered optimal discriminatory power with respect to the diagnosis of Major Depressive Disorder via the clinical interview (sensitivity of 86% and a specificity of 67%) [28]. In line with this, in another validation study of the PHQ-9 involving HIV-positive patients conducted in Jimma, South-Western Ethiopia, a cutoff score of 6 was found to have a sensitivity of 87.2 and specificity of 83.7 for the presence of major depression [29]. Another validation study done in Ethiopia PHQ-9 has a sensitivity of 77.8, a specificity of 80.6, and a negative predictive value of 98.3 at a cutoff score of 6 or more [30]. Therefore, in this study, participants scoring six or more were classified as having probable depression. The already validated Amharic translation of PHQ-9 was employed in this study. According to the PHQ-9 score categorization, <6, 6–10, 10–15, 15–20, > = 20 had no/minimal, mild, moderate, moderately severe, and severe depression, respectively.

#### Adherence measurement

The adherence level was measured using the Center for Adherence Support Evaluation (CASE) Adherence Index, which is freely available to assess ART adherence in a clinical setting [31]. The index is comprised of three self-reported measures of adherence: self-reported frequency of ‘difficulty taking HIV medications on time (no more than two hours before or two hours after the time your doctor told you to take it)’(responses were: ‘never, rarely, most of the time or all of the time’); self-reported ‘an average number of days per week at least one dose of HIV medications was missed’ (responses were: ‘every day, 4–6 days per week, 2–3 days per week, once a week, less than once a week or never; reverse coded for analysis’); and self-reported ‘last time missed at least one dose of HIV medications’ (responses were: ‘within the past week, 1–2 weeks ago, 3–4 weeks ago, between one and three months ago, more than three months ago or never’).

A woman was considered to have good adherence if the CASE index score was > 10, and she was considered to have ‘poor adherence’ if the index score ≤ 10.

### Data collection and quality assurance

Trained data collectors with supervision from the principal investigator (PI) collected the data. The psychiatrist (co-investigator) provided additional oversight to ensure data quality. ANC providers (including nurses, health officers, and physicians) in a given study facility informed HIV-positive women in their third trimester about the study and referred them to the study nurse if they were interested in participating. After confirming the eligibility, the study nurse obtained written consent. An interview was then conducted to collect socio-demographic and clinical information, including screening for depression and adherence to ART. Mid-Upper Arm Circumference (MUAC) with the cutoff less than or equal to 23cms was used for nutritional assessment; CD4 and viral load measurements had been performed within three months of the data abstraction.

### Data analysis

Before analysis, the completeness of the data was evaluated. We used Statistical Package for the Social Sciences (SPSS) version 24 for data entry and analysis. Descriptive statistics was employed to estimate the prevalence of depression during third-trimester pregnancy and nonadherence to ART. Bivariate logistic regression analyses was employed to get significant predictors for each of the two outcome measures. Age, educational status, marital status, and variables with a P value less than 0.25 in the bivariate logistic regression model were included in the final multivariable logistic regression analysis. All statistical significance was evaluated at *P*<0.05.

### Ethical considerations

Ethical approval was obtained from the Institutional Review Board (IRB) of the College of Health Sciences, Addis Ababa University, Addis Ababa Health Bureau (AAHB) (Protocol number075/17/Ped), and Johns Hopkins University Bloomberg School of Public Health (IRB No: IRB00008023). Written voluntary consent was obtained from participants who agreed to participate in the study. Interviews were held in private consultation rooms to maintain confidentiality and privacy. The study team was trained to obtain information from the study participants or their medical records without identifiers. We ensured mental health professional diagnostic assessment and treatment for women with a PHQ-9 score of ≥ 6 and/or suicidal ideation. Enhanced adherence counseling was provided for those participants who had inadequate adherence.

## Results

### Socio-demographic and economic characteristics of participants

A total of 368 women (92.7%) accepted to participate in the study out of a total of 397. The women ranged in age from 20 to 45 years, with a mean age of 30.79 (SD = 4.7). Out of the total 368 women included in this study, 308(83.3%) were currently married, about half, 183(49.7%) had attended primary education or below, and 318 (86.4%) were Orthodox Christians, and 50 (13.6%) were Muslims. The majority, 341 (92.7%), were residents in Addis Ababa, and three-fourths (292, 79.3%) were living within less than a 10 km radius from the health facility. More than half of the participants, 196 (53.3%), were unemployed (housewives), and 199 (54.1%) had a monthly income of less than or equal to 2500 Ethiopian Birr (89$). Nearly three-quarters of pregnant women (191; 48.6%) were multigravidas (Table 1).

**Table 1:** Bivariate associations of depression and nonadherence to ART with sociodemographic and clinical characteristics of pregnant women living with HIV, 2018, Addis Ababa. (*n = 368*).

Characteristics of Pregnant Women	N (%)	Depressed N (%)	p-value	Nonadherence N (%)	p-value
Age (Years)					
20–29	153(41.6)	69(45.1)	0.426	29(18.9)	0.667
30 and above	215(58.4)	106(49.3)		37(17.2)	
Educational Status					
Primary school and below	183(49.7)	93(50.8)	0.213	35(19.1)	0.554
High School and above	185(50.3)	82(44.3)		31(16.8)	
Marital status					
Single	60(16.3)	30(50)	0.679	9(15.0)	0.518
Currently Married	308(83.7)	145(47.1)		57(18.5)	
Religion					
Christian	318(86.4)	152(47.8)	0.813	55(17.3)	0.422
Muslim	50(13.6)	23(46)		11(22)	
Occupation					
Unemployed	196(53.3)	98(50)	0.316	45(22.9)	**0.008**
Formally or self-employed	172(46.7)	77(44.8)		21(12.2)	
Monthly Income in Ethiopian Birr^a^($)					
≤ 2500 (89)	199(54.1)	110(55.3)	**0.001**	37(18.6)	0.721
>2500 (89)	169(45.9)	65(38.5)		29(17.2)	
Distance from Health Facility					
< = 10kms	292(79.3)	141(48.3)	0.581	54(18.5)	0.585
>10kms	76(20.3)	34(44.7)		12(15.8)	
Children					
Yes	266(72.3)	135(50.8)	0.048	47(17.7)	0.830
No	102(27.7)	40(39.2)		19(18.6)	
Mid Upper Arm Circumference (MUAC)					
< = 23	62(16.8)	28(45.2)	0.679	11(17.7)	0.965
>23	306(83.2)	147(48)		55(18)	
CD4 cells/mm^3^ (n = 352)					
< = 350	80(22.7)	32(40.0)	0.162	14(17.5)	0.779
>350	272(77.3)	133(48.9)		44(16.2)	
WHO Clinical Stage					
Stage 1	342(92.9)	153(44.7)	**0.001**	61(17.8)	0.858
Other Stages	26(7.1)	22(84.6)		5(19.2)	
ART regimen					
TDF/3TC/EFV	261(71.3)	122(46.7)	0.627	42(16.1)	0.152
Other regimens	107(28.9)	53(49.5)		23(21.5)	
Depression					
Nondepressed	193(52.4)			26(13.5)	**0.020**
Depression	175(47.6)			40(22.9)	

^a^1 USD($) = 28ET at the time of the study.

*P value <0.05, significantly associated. CI: Confidence interval, OR: Odds ratio.

### Clinical and behavioral characteristics of participants

The majority of the participants, 342(92.9%), were at WHO HIV Clinical Stage 1. Sixty-two (16.8%) had mid-upper arm circumference (MUAC) of less than or equal 23cms. The majority, 272 (73.9%) of the participants, had a CD4 count of greater than 350, with a median count of 480. Most of the participants, 304 (82.6%), had a hemoglobin of greater than 11gm/dl with mean hemoglobin of 12.5 (SD = 1.3, range from 8.5–15.9). Half of the participants were taking TDF, 3TC, EVF as their ART regimen (Table 1).

Comorbid conditions like diabetes mellitus, hypertension, or chronic obstructive lung disease (COPD) were seen in only 38 (10.3%) cases. Only one reported smoking cigarettes, 25 (6.8%) reported ever-chewing chat, and 35 (9.5%) reported drinking alcohol during this pregnancy.

### Prevalence and predictors of depression

A total of 175 (47.6%) study participants had depression using the PHQ-9 score cutoff of ≥ 6 (Table 1). According to the PHQ-9 score categorization, 78(21.2%), 63 (17.1%), 23 (6.3%), and 11 (3.0%) had mild, moderate, moderately severe, and severe depression, respectively. Income was significantly associated with depression using multivariate analysis. Women with a monthly income less than or equal to 2500 Birr were twice (AOR = 2.10, 95% CI = 1.31–3.36) as likely to have depression compared to those with a monthly income greater than 2500 birrs. Participants with WHO HIV Clinical Stage I (AOR = 0.16, 95% CI = 0.05–0.48) were less likely to have depression than women with advanced stages. Pregnant women who reported having children were more likely to be depressed (AOR = 1.81, 95% CI = 1.02–3.23) (Table 2).

**Table 2:** Multivariable logistic regression analysis of factors associated with depression among pregnant women living with HIV, Addis Ababa, 2018. *(n = 368)*.

Characteristics of Pregnant Women		Depression		Adjusted OR (95%CI)
		Non-depressed (%)	Depressed (%)	
Age (Years)	20–29 30 and above	84(54.9) 109(50.7)	69(45.1) 106(49.3)	1.15(0.71, 1.88) 1
Educational Status	Primary school and above High School and Above	90(49.2) 103(55.7)	93(50.8) 82(44.3)	0.93(0.59,1.48) 1
Marital status	Single Currently Married	30(50) 163(52.9)	30(50) 145(47.1)	0.85(0.45,1.64) 1
Monthly Income (Birr)	< = 2500 >2500	89(80.9) 104(61.5)	110(55.3) 65(38.5)	**2.10(1.31,3.36)** 1
No of Children	Yes No	131(49.2) 62(60.8)	135(50.8) 40(39.2)	**1.81(1.02,3.23)** 1
CD4 cells/mm^3^ (n = 352)	< = 350 >350	48(60.0) 139(51.1)	32(40.0) 133(48.9)	1.47(0.85,2.53) 1
WHO Clinical Stage	Stage 1 Other Stages	189(55.3) 4(15.4)	153(44.7) 22(84.6)	**0.16(0.05,0.48)** 1

AOR: Adjusted Odds Ratio, CI: Confidence Interval.

### Depression and adherence to ART

Eighty-two percent (95%CI 78–86%) of participants have good adherence to their ART. There was a statistically significant association between depression and nonadherence to ART (P = 0.025). Study participants who were depressed were nearly two times (AOR = 1.96, 95%CI = 1.03–3.75) more likely to be non-adherent to ART compared to study participants with no depression (Table 3).

**Table 3:** Multivariable logistic regression analysis of factors associated with adherence among pregnant women living with HIV, Addis Ababa, 2018. *(n = 368)*.

Characteristics of Pregnant Women		Adherence		Adjusted OR (95%CI)
		Adherent (%)	Nonadherent (%)	
Age (Years)	20–29 30 and above	124(81.0) 178(82.8)	29(19) 37(17.2)	0.79(0.45, 1.38) 1
Educational Status	Primary school and above High School and Above	148(80.9) 154(83.2)	35(19.1) 31(16.8)	1.01(0.58,1.78) 1
Marital status	Single Currently Married	51(85) 251(81.5)	9(15.0) 57(18.5)	1.27(0.57,2.84) 1
Occupation	Unemployed Formally or self-employed	151(77) 151(87.8)	45(23) 21(12.2)	**2.09(1.16,3.74)** 1
ART regimen	TDF/3TC/EFV Other regimens	219(83.9) 84(78.5)	42(16.1) 23(21.5)	0.65(0.36,1.16) 1
Depression	Depressed Nondepressed	135(77.1) 167(86.5)	40(22.9) 26(13.5)	**1.88(1.08,3.27)** 1

AOR: Adjusted Odds Ratio, CI: Confidence Interval.

## Discussion

In this study, which was conducted in public health facilities of Addis Ababa, we found high prevalence of depression among HIV-positive pregnant women, and it was associated with nonadherence to ART. Availability of highly active antiretroviral treatment worldwide has transformed HIV from a terminal acute disease to a chronic disease with the prospect of long survival. People living with HIV need to be monitored for comorbid psychiatric conditions, including depression [32]. Socio-demographic and clinical characteristics appeared associated with depression, some of which are potentially amenable to change with intervention.

In our study, the prevalence of depression of 46.7% is comparable with two reports from South Africa [33] and pooled prevalence report in systematic reviews [34]. On the other hand, it is slightly higher than reports from Zimbabwe from Africa and Ukraine [35,36]. Depression is more prevalent among HIV-positive pregnant women than HIV-negative pregnant women as reported from Gondar, northern Ethiopia [37]. The prevalence of depression among HIV-positive pregnant women varies from place to place, which may be due to a real difference in magnitude or diagnostic tool used. The prevalence of depression observed in this study was among the higher rates seen in the sub-Saharan Africa region, indicating a need to better screen and identify a condition that could adversely affect overall maternal and infant outcome.

Income was significantly associated with depression after controlling for the other confounding factors. Financial constraints impairing a woman’s ability to address her family’s basic needs could affect their mental health. In addition, pregnancy may decrease their employability and even their potential to work because of the type of labor impoverished women may need to undertake [38]. We cannot be sure that the disability from depression is the cause for low income as it is difficult to determine the direction of causality from cross-sectional studies [39]. Studies from high-income countries such as Australia and the USA reported similar results regarding the association of depression with low income [40,41]. In this study, pregnant women who had one or more children were depressed compared to those with none. The family’s additional economic and time burden might explain why women with a child have depression [42].

Our results also indicate a significant association between depression and stage of HIV disease, the women with advanced WHO stage of HIV disease had a higher risk of having depression than those at the early stages of HIV. This finding is consistent with a review of several studies that showed higher rates of depression with the advanced stage of HIV disease [43]. The prevalence of depression was also seen to increase in tandem with the stages of HIV diseases progression [44]. Preventing disease progression is essential by promoting HIV care and treatment, mainly antiretroviral treatment, which potentially contributes to preventing depression.

Provision of ART to all HIV-infected pregnant women is the primary modality of preventing MTCT, and reduction of maternal viral load to an undetectable level reduces the risk of transmission to the newborn close to zero [45]. Achievement of viral suppression requires an adherence level of at least 95% [46]. However, our finding was that 82% of HIV-positive pregnant women had good adherence to ART, lower than the required adherence level for viral suppressions. The adherence level in our study, though lower than the studies done in Tigray Ethiopia and western Kenya [47,48], is comparable to that reported from several other African countries like Kenya, Nigeria, Uganda, Malawi, and also with a pooled estimate for LMICs [6,10,49–52]. Low medication adherence to lifelong treatment is not uncommon; it has been observed in adult and children individuals living with HIV [53–55], pregnant women with diabetes mellitus [56], and people with mental health problems[57].

The results of this study revealed that those who were depressed were nearly twice as likely to be non-adherent to their ART. In general, a desire to protect an unborn baby from acquiring HIV is a strong motivator for good adherence to preventive medication. However, loss of interest, hopelessness, and fatigue, which are symptoms of depression, maybe reasons for poor adherence to ART, as reported by other studies [8,9]. Proper treatment and alleviation of symptoms of depression are vital to improve adherence to ART [24] and to optimize the woman’s health and that of her unborn child.

### Limitations

The cross-sectional design of this study makes it difficult to determine the direction of association. The tools also had limitations. Although the PHQ-9 is standard and validated for screening depression in Ethiopia by non-professionals who are trained to screen and identify with depression, it does not provide a definitive diagnosis of depression as defined by either the DSM-5 or the International Classification of Disease version 10 (ICD-10). Even though the CASE adherence tool is easy to administer it suffers from the social desirability bias as health professionals administered it.

## Conclusion

We found high prevalence of depression among HIV-positive pregnant women in Addis Ababa. Low income was associated with antenatal depression in HIV-positive pregnant women. Our finding also showed that adherence to ART among depressed women was lower than those who were not depressed. Routine screening and management of depression for HIV-positive pregnant mothers attending ANC is recommended. There is also a need to promote enhanced adherence counseling for those depressed pregnant women since poor adherence increases the risk of MTCT.

## Supporting information

S1 File Depression Fogarty latest. (SAV)

S2 File Questionnaire depression study. (PDF)

## Abbreviations

AIDS: Acquired Immunodeficiency Syndrome
ANC: Antenatal Care
ART: Antiretroviral therapy
EID: Early Infant Diagnosis
EPDS: Edinburgh Postnatal Depression Scale
HIV: Human Immunodeficiency Virus
MCH: Maternal and child health
MOH: Ministry of Health
MTCT: Mother to Child Transmission of HIV
PMTCT: Prevention of Mother To Child Transmission
WHO: World Health Organization
