# Supplementary material for: Prevalence of depression among HIV-positive pregnant women and its association with adherence to antiretroviral therapy in Addis Ababa, Ethiopia
Source: PLoS One. 2022 Jan 20;17(1):e0262638. doi: 10.1371/journal.pone.0262638 (PMC8775187; doi:10.1371/journal.pone.0262638)
Supplement: S2 File — (PDF) [file pone.0262638.s002.pdf]

Annex -2

Participant Identification Number: __ __ __ __ __/__ _

Prevalence of antenatal depression pregnant women and its association with adherence to ART among HIV-infected women in Addis Ababa, Ethiopia

**Section 1: Demographic Information**		
**Question**	**Response**	**Code**
**101**. How **old** are you?	Years |__|__|	
**102.** What is the **Highest level of education** you have completed?	No formal schooling 1<br>Less than Primary School completed 2<br>Secondary school Completed 3<br>College and Above 4<br>No information 5<br>Refused 88	
**103.** Place of **Residence**	Urban 1<br>Rural 2	
**104.** What is your **Religion**?	Orthodox 1<br>Protestant 2<br>Muslim 3<br>Catholic 4<br>---------------------------Other (specify) 5	
**105.** What is your **Marital status**?	Never married 1<br>Currently married 2<br>Separated 3<br>Divorced 4<br>Cohabitating 5<br>Refused 88	
**106.** Which of the following best describes your **main employment** status over the past 12 months?	Government employee 1<br>Non-Government employee 2<br>Private employee 3<br>Student 4<br>Unemployed 5<br>Refused 88<br>---------------------------Other (specify) 5	
**107.** Taking the past year, can you tell me what the average expenses (Birr) of the household have been?<br><br>RECORD ONLY ONE, NOT ALL 3	Per week __ __ __ __ __ __ __ __ __<br>Or Per month __ __ __ __ __ __ __ __<br>Or Per Year __ __ __ __ __ __ __ __<br>Refused 88	
**108.** How many **children** do you have?	Number __ __	
**109.** Distance from home to **health facility**?	Kms __ __<br>Or<br>Mention the Place--------------------------------	

Section 2: Clinical Characteristics		
**Question**	**Response**	**Code**
**201.** Date HIV tested?	-------/-------/-------- (DD/MM/YY)	
**202.** Date since ART was started?	-------/-------/-------- (DD/MM/YY)	
**203.** Body weight	-----------kg	
**204.** Height	-----------mts	
**205.** MUAC	-----------cms	
**206.** HGB/HCT	-----------	
**207.** Current CD4 Count	-------------(Count/ml)	
**208.** Total Lymphocyte Count	------------------ (Count/ml	
**209.** Viral Load	------------------Copies /ml	
**210.** WHO clinical stage	Stage I 1<br>Stage II 2<br>Stage III 3<br>Stage IV 4	
**211.** Functional Status	Working 1<br>Ambulatory 2<br>Bed ridden 3<br>Not recorded 4	
**212.** Initial ART Regimen	1e 1<br>1f 2<br>1c 3<br>1d 4<br>Other ______________________________ 5	
**212.** If the patient is on second line	Mention the regimen__________________________	
**213.** Number ANC visits	Number __ __	
**214.** Any additional diagnosis	Yes 1<br>No 2<br>If Yes, Specify _<br>____________________	
**215.** Any additional treatment other than ART	Yes 1<br>No 2<br>If Yes, Specify _<br>____________________	
**216**. Do you drink alcohol (such as beer, liquor, arake, tej) or use any psychoactive substance (such as khat, tobacco, shisha, hashish) during this pregnancy?	Yes 1<br>No 2<br>If Yes, Specify _	

ክፍል 3፡ የፀረ ኤችአይቪ መድሃኒትን ያለማቋረጥ ስለመከታተል		
ጥያቄ	ምላሽ	ኮድ
301. ምን ያህል ጊዜ የኤችአይቪ መድሃኒትዎን በወቅቱ ለመውሰድ አስቸጋሪ ሆኖ ይሰማዎታል? "በወቅቱ" የሚለው ምላሽ ሃኪምዎ መድሃኒቱን እንዲወስዱ ከነገርዎት ሰአት ከ2 ሰአት በፊት ወይም በኋላ ሳልበለጠ ጊዜ ማለት ነው)፡፡	ሁልጊዜ 1<br>አብዛኛውን ጊዜ 2<br>አልፎ አልፎ 3<br>ፈጽሞ አያስቸግረኝም 4	
302. በአማካኝ ከሳምንቱ ቀናት ውስጥ ምን ያህል ቀናት ቢያንስ አንድ ጊዜ የኤችአይቪ መድሃኒትዎን ለመውሰድ ይረሳሉ?	ቀን በቀን 1<br>4-6 ቀናቶች 2<br>2-3 ቀናቶች/ሳምንት 3<br>አንድ ቀን በሳምንት 4<br>በሳምንት ከአንድ ጊዜ በታች 5<br>ፈጽሞ አልረሳም 6	
303. ቢያንስ አንድ ጊዜ የኤችአይቪ መድሃኒትዎን ለመውሰድ ለመጨረሻ ጊዜ የረሱት መቼ ነው?	ባለፈው ሳምንት ውስጥ 1<br>1-2 ሳምንት በፊት 2<br>3-4 ሳምንት በፊት 3<br>ከ1-3 ወር በፊት 4<br>ከ3 ወር በላይ 5<br>ፈጽሞ አልረሳም 6	

ክፍል 4፡ የፀረ ኤችአይቪ ኤዲስ መድሃኒት ደካማ የሆነ አወሳሰድ ምክንያት	
ጥያቄ	ምላሽ
401. ሰዎች በተለያዩ ምክንያቶች መድሃኒታቸውን መውሰድ ሊረሱ ይችላሉ፡፡ ባለፈው ወር የፀረ ኤችአይቪ መድሃኒትዎን ያልወሰዱበት ምክንያቶች ምን ምን ናቸው? ከአንድ በላይ መልስ መስጠት ይቻላል፡፡	ከቤት በመራቅ ምክንያት 1<br>በሌሎች ጉዳዮች መጠመድ 2<br>በመርሳት ብቻ 3<br>ብዙ መድሃኒት በመውሰድ ምክንያት 4<br>አሉታዊ የግንኙነቶች ውጤት እንዳይደርስ 5<br>ሌሎች መድሃኒቱን ስውስድ እንዲያዩኝ ስለማልፈግ 6<br>በእለታዊ ስራ ለውጥ ምክንያት 7<br>መድሃኒቱ ጉጂ መስሎ ስለሚሰጠኝ 8<br>የመታመም ስሜት ስለሚሰማኝ 9<br>የድብርት ስሜት ስለሚሰማኝ 10<br>መድሃኒቱ ስላለቀብኝ 11<br>መድሃኒቱን ሳልወስድ ስቀር ጥሩ ስሜት ይሰማኛል 12<br>የትራንስፖርት አለመኖር 13<br>የምግብ እጥረት 14<br>……….. ሌሎች<br>ግለጽ 15
